# Relationship between Muscle Cross-Sectional Area by MRI and Muscle Thickness by Ultrasonography of the Triceps Surae in the Sitting Position

**DOI:** 10.3390/healthcare8020166

**Published:** 2020-06-10

**Authors:** Ryo Miyachi, Toshiaki Yamazaki, Naoki Ohno, Tosiaki Miyati

**Affiliations:** 1 Department of Physical Therapy Faculty of Health Science, Kyoto Tachibana University, Kyoto 607-8175, Japan; miyachi@tachibana-u.ac.jp; 2 Faculty of Health Sciences, Institute of Medical, Pharmaceutical and Health Sciences, Kanazawa University, Kanazawa 920-0942, Japan; nohno@med.kanazawa-u.ac.jp (N.O.); ramiyati@mhs.mp.kanazawa-u.ac.jp (T.M.)

**Keywords:** triceps surae, MRI, sitting position

## Abstract

The purpose of this study was to determine the differences in the muscle cross-sectional area (MCSA) of the triceps surae in the supine and sitting positions using magnetic resonance imaging (MRI), and the relationship between the MCSA of the triceps surae in the sitting position and muscle thickness (MT), assessed using MRI and ultrasonography, respectively. This study included 16 healthy young male participants. The measurement positions were 90° flexion of the knee joint and neutral position of the ankle joint in the sitting or supine positions. Using an open-configuration MRI system with a vertical gap and ultrasonography, we measured the MCSA and MT of the soleus muscle and the medial and lateral heads of the gastrocnemius muscle at three selected locations in the ventral part of the muscle. As a result, the 50% portion of the soleus muscle and the 25% and 50% portions of the gastrocnemius medial and lateral heads were higher in the sitting position than in the supine position. Furthermore, only 50% of the gastrocnemius medial head showed a correlation between the MCSA and MT. When using the MT of the triceps surae as an indicator of muscle volume in the sitting position, the muscle site should be considered.

## 1. Introduction

The triceps surae is the major ankle plantar flexor muscle. It is said that the triceps surae is involved in several movements such as postural control, walking, running, and jumping and has an effect not only on the ankle joint but also on the proximal joint [1,2,3]. It is important to maintain and strengthen the triceps surae because a weakened triceps surae increases the risk of falling and interferes with several activities of daily living [4]. The relationship between muscle strength and volume is well known, and the circumference of the lower leg is often measured as a rough indicator of muscle volume. However, this circumference is affected by several factors such as fat, bone, and nerves, and therefore, is not an accurate measurement of muscle volume. Hence, the importance of evaluating muscle volume using images has been indicated [5]. The muscle cross-sectional area (MCSA) assessed using magnetic resonance imaging (MRI) and muscle thickness (MT) determined from ultrasonography (US) are often used as nonexplosive and noninvasive methods to measure the muscle volume from images. Physiological MCSA perpendicular to the muscle fibers and anatomical MCSA perpendicular to the long axis of the entire muscle have been reported to be related to muscle strength [6,7,8], and the MCSA has been reported to be a useful indicator of muscle strength [9]. Previous studies have reported that muscle morphology in the lying position is affected by muscle deflection and compression, and the MCSA and MT measured in the lying position are lower than in the standing position [10,11]. Conventional MRI is generally limited to the lying position only because of the structure of the equipment. However, the recently developed open-configuration MRI system with a vertical gap (vertical MRI) can be used in any limb position [12,13]. To obtain basic information on the MCSA in the lower leg muscles in the sitting position, we analyzed and examined the MCSA in the sitting position using vertical MRI [14]. However, our previous study only measured the sitting position and did not investigate the difference in the MCSA between the lying and sitting positions. In addition, although vertical MRI can provide clear, wide-area images for MCSA measurements, its major disadvantages are its rarity and lack of ease of imaging. US is widely used as a safe and easy method for image evaluation in several facilities. The US evaluation of triceps surae thickness has a high interclass correlation coefficient and is reported to be reliable [9,15,16,17]. On the other hand, US evaluation requires proficiency in probe operation and image evaluation [18], and it is challenging to obtain more than two-dimensional values when measuring muscle volume because of the difficulty in evaluating a wide area. Therefore, there are few studies that have examined the correlation between the MT evaluated by US and MCSA in previous studies [19,20], but none have examined the antigravity position. If MT determined by US in the antigravity position is associated with MCSA obtained by MRI, then MT can be used in the clinical evaluation of the triceps surae muscle volume in the antigravity position.

The purpose of this study was to compare the MCSA of the triceps surae determined by MRI in the lying and sitting positions and investigate its relationship with MT assessed using US in the antigravity position to establish a simple method to obtain information on the muscle volume of the triceps surae.

## 2. Materials and Methods

### 2.1. Participants

This study included 16 healthy young male participants without any pain reported in their activities of daily life; more specifically, it focused on the dominant leg that the participants would use to kick a ball. The mean (± standard deviation) age, height, weight, and lower leg length (fibular head to lateral malleolus) were 20.9 ± 1.4 years, 169.2 ± 3.7 cm, 61.1 ± 6.1 kg, and 33.1 ± 1.5 cm, respectively. None of the participants had a history of orthopedic problems of the legs or the vertebrae.

The protocol for this study was approved by the Ethics Committee of Kanazawa University (approval number: 687). Before conducting the study, we explained the objective and content of the study to the participants and informed them that the data obtained from this study would not be used for any purpose other than this study, assuring them that the data would be handled strictly in confidence to prevent the dissemination of personal information. Written informed consent was obtained from all participants before the study.

### 2.2. Measurement

**Figure 1:**
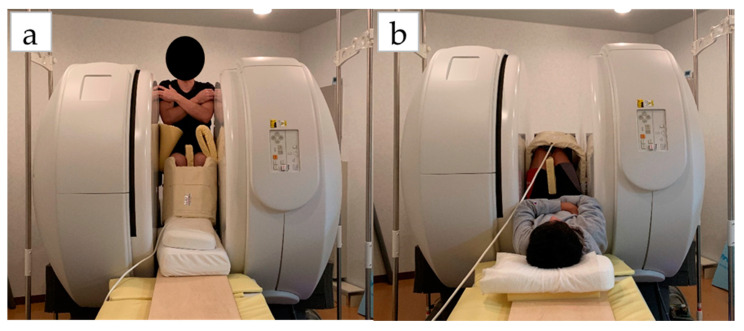
Measurement of position on vertical MRI: (**a**) sitting position; (**b**) lying position.

**Figure 2:**
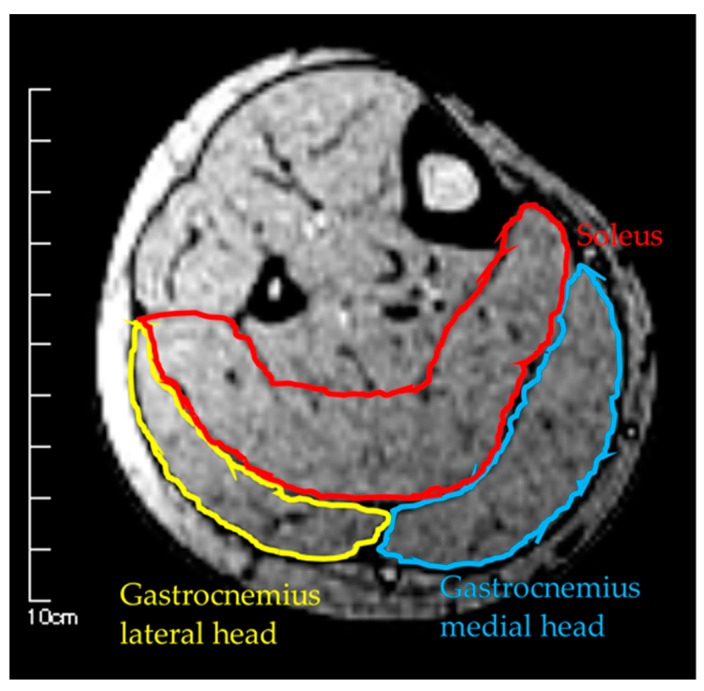
Measurement of muscle cross-sectional area by MRI.

**Figure 3:**
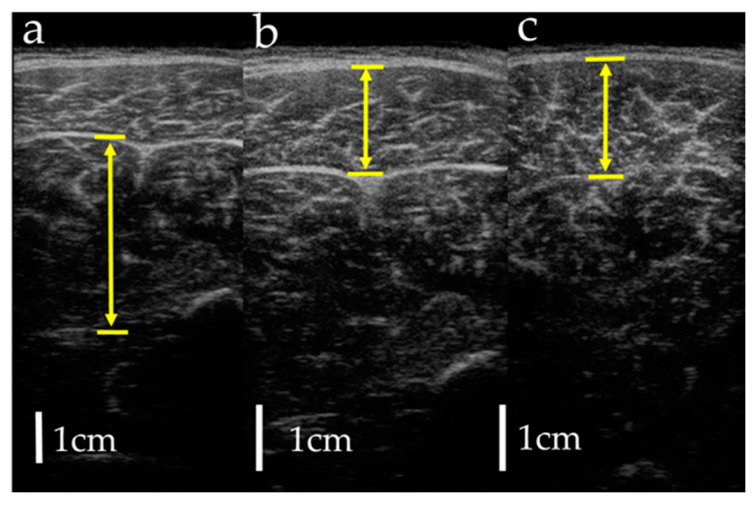
Measurement of muscle thickness by ultrasonography: (**a**) soleus; (**b**) gastrocnemius lateral head; (**c**) gastrocnemius medial head. Arrows indicate muscle thickness.

Measurements were taken in three conditions: (1) MRI in the supine position, (2) MRI in the sitting position, and (3) US imaging in the sitting position. The sitting position was chosen for the measurement of the antigravity position because it is easy to hold the limb position with little motion in the antigravity position. In the supine position for MRI, the foot was placed on a pedestal in a supine position in which the hip and knee joints were flexed at 90°, and the foot was supported only by the heel to prevent the triceps surae from changing its shape due to contact with the pedestal. In addition, a plate was placed perpendicular to the floor at the plantar part of the foot to maintain dorsiflexion at 0°. MRI and US imaging in the sitting position were performed in a chair (with a backrest) with the knee joint flexion at 90°, the lower leg perpendicular to the floor, and the ankle joint dorsiflexion at 0° as in the supine position. In all measurements, the thigh was fixed with cushions and bands to prevent the lower leg from moving during the imaging, and the participants were instructed to hold it as much as possible. Using vertical MRI with a 0.4 T permanent magnet, (Hitachi Healthcare, Ltd., Tokyo, Japan), horizontal T1-weighted images were obtained from the fibular head to the 290 mm distal end at 10 mm intervals (Figure 1). The imaging parameters were as follows: slice plane axial; pulse sequence RF-spoiled steady-state gradient echo; field of view, 280 mm; repetition time, 110.0 ms; echo time, 8.6 ms; flip angle, 35°; slice thickness, 10.0 mm; slice interval, 10.0 mm; matrix size, 256 × 256; number of signals, average of 2; receiver bandwidth, 20.6 kHz; and scan time, 4 min and 32 s. Using the image analysis program ZedView (LEXI Co., Ltd., Tokyo, Japan), the muscles were identified based on the boundary of the fascia of the soleus (SOL), gastrocnemius medial head (GM), and gastrocnemius lateral head (GL) using the acquired images, and the areas marked on the images were measured as the MCSA of each muscle (Figure 2). The 10 mm slice areas of MRI were summed, and the volume of each muscle was calculated using a ZedView. The images of the SOL, GM, and GL were taken by US using a linear probe (7.5 MHz) in B-mode MyLab25 (Esaote, Florence, Italy). Short-axis images were taken at 20.0 mm intervals from the peroneal head in each muscle by applying a probe to the center of the ventral region of the muscle on the horizontal plane. We used a hard-type gel (LOGIQLEAN, GE Healthcare Japan, Tokyo, Japan), which is flexible and easy to keep in shape, to avoid direct contact between the probe and the lower leg. Indirect contact through the gel avoids the effects of pressure exerted by the probe on the lower leg. In addition, to maintain the position of the probe at the time of measurement, we marked the lower leg prior to the measurement, following which assessments were made based on the marking. For the measurement of MT, the maximum length of each muscle was measured with respect to the boundary of the fascia using the image analysis program Image J (Figure 3). The average value of the two measurements was calculated and used as the MT value. MRI, US imaging, and MCSA and MT measurements were performed by the same examiner.

### 2.3. Statistical Analysis

**Figure 4:**
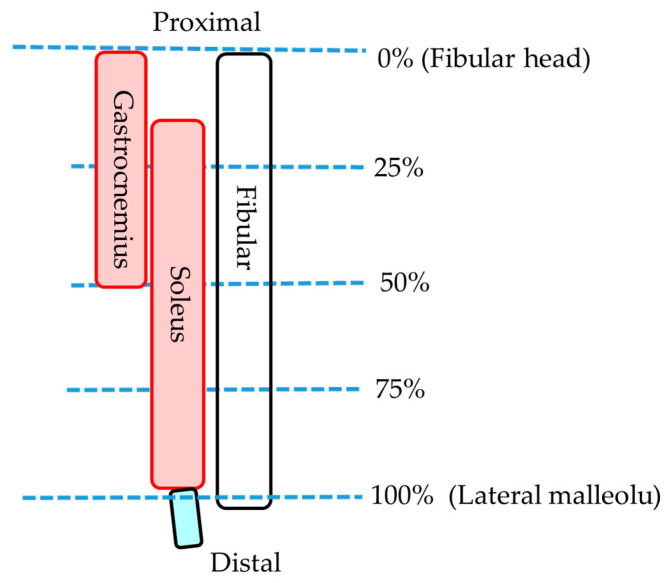
Measurement portion of each muscle.

Statistical analyses were performed using SPSS Ver. 24 (IBM SPSS Statistics, Japan IBM, Tokyo, Japan). For statistical analysis, three portions were selected: 25%, 50%, and 75% (0% is fibular head and 100% is lateral malleolus) of the muscle (Figure 4). The lower leg length of the participant with the longest lower leg length was 35.0 cm; therefore, all participants were able to obtain 75% portion image by MRI. *p* values < 0.05 were considered significant. After confirming the normality of the data with the Shapiro–Wilk test, paired t-test was used for the comparison of the MCSA in the lying and sitting positions and muscle volume on the MRI. Pearson’s correlation coefficients were used to determine the relationship between MCSA and MT. One-way analysis of variance and the Bonferroni test were used to compare the MCSA in the same region, the volume of each muscle, and the MCSA in each region in the same muscle in the lying position. All measurements are presented as the mean ± standard deviation.

## 3. Results

**Figure 5:**
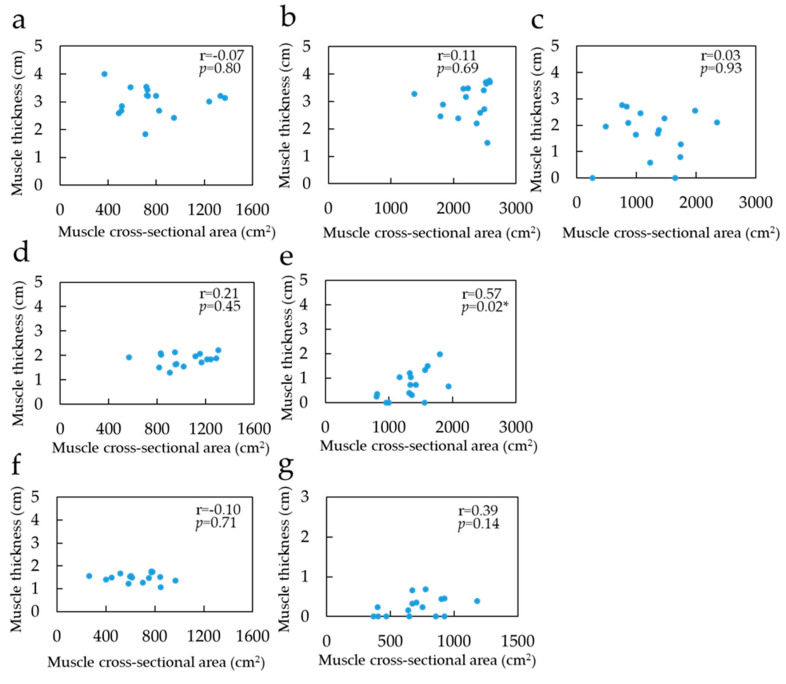
Correlation coefficient between muscle cross-sectional area in sitting position and muscle thickness: (**a**) Soleus of 25% portion; (**b**) Soleus of 50% portion; (**c**) Soleus of 75% portion; (**d**) Gastrocnemius medial head of 25% portion; (**e**) Gastrocnemius medial head of 50%; (**f**) Gastrocnemius lateral head of 25% portion; (**g**) Gastrocnemius lateral head of 50% portion. The blue point indicates value of muscle thickness and muscle cross-sectional area. * Significant correlation with the muscle cross-sectional area on MRI (*p* < 0.05).

Table 1, Table 2 and Table 3 show the MCSA in the lying and sitting positions, MT, and muscle volume for each muscle, respectively. Furthermore, 75% portions of the GM and GL were excluded from the statistical analysis because of the absence of muscle belly. On comparing the MCSA in the lying and sitting positions, the 50% portion of the SOL was found to be significantly greater in the sitting position than in the lying position. Both 25% and 50% portions of the GM were significantly greater in the sitting position than in the lying position. However, no significant difference was observed in the MCSA between the lying and sitting positions in either the 25% or the 50% portions of the GL. Furthermore, no significant difference was found in the muscle volume between the lying and sitting positions in all muscles. The relationship between MCSA and MT in the sitting position was not correlated with SOL and GL (Figure 5). However, for GM, there was a significant correlation, albeit only in the 50% portion (*p* < 0.05, r = 0.57). In the comparison of the MCSA in each muscle in the same region in the lying position, the 25% portion of the GM and SOL was significantly greater than that of the GL. In addition, the 50% portion of SOL, GM, and GL was significantly large. Muscle volume in the lying position was significantly high in SOL, GM, and GL, in that order. Comparisons of MCSA in the lying position between the regions indicated that the SOL was significantly high in the 50%, 25%, and 75% portions in that order. As for the GM, the measurement was significantly greater in the 50% portion than in the 25% portion. There was no significant difference in the GL between the 25% and 50% portions.

## 4. Discussion

The purpose of this study was to compare the MCSA of the triceps surae in the lying and sitting positions measured using vertical MRI and clarify the differences in the MCSA depending on the posture. In addition, we aimed to clarify the relationship between the MT measured using US, which is a commonly used instrument, and MCSA, and use the MT as an indicator of MCSA in the sitting position. The results of this study are important as an indicator of the structural factors related to muscle strength, such as the measurement of leg circumference and MT measured by US in the antigravity limb position.

There was no difference in the muscle volumes between the lying and sitting positions, suggesting that there was no change in the muscle volumes due to posture, and either the lying or sitting position did not under- or over-measure the muscle. On the other hand, for the MCSA, as in previous studies [10,11], the difference was greater in the sitting position than in the lying position. Kinugasa et al. [21] reported that the MCSA decreases when the muscle is stretched in the longitudinal direction because the muscle volume is constant. In the present study, in the sitting position, the MCSA increased in the area where the muscles moved caudally due to the effect of gravity, while it decreased in some areas due to stretching. In the SOL, there was no difference in the MCSA between the lying and sitting positions at the 25% portion, though the MCSA in the sitting position was greater at the 50% portion. The 25% portion was located more proximally than the 50% portion; therefore, the amount of muscle that could be moved caudally was expected to be less than in the 50% portion when the whole muscle belly was considered. Furthermore, the MCSA in the 25% portion was lower than that in the 50% portion, suggesting that the muscle volume in the 25% portion was smaller than that in the 50% portion and less susceptible to the effects of gravity. Similarly, the MCSA in the 75% portion was smaller than that in the 50% portion, suggesting that the area was less susceptible to muscular deflection. For the GM, the MCSA in the sitting position was greater than in the lying position in both 25% and 50% portions, suggesting that the GM is more susceptible to postural changes than the SOL in the whole muscle. It is considered that the GM is more susceptible to gravitational deflection than the SOL because it is located in the superficial region. Furthermore, there was no difference in the MCSA between the lying and sitting positions for the GL located in the superficial region as well as for the GM. It has been reported that the muscle strength of the GL is smaller than that of the GM [1]. In the present study, the MCSA of the GL was smaller than that of the SOL and GM in both the 25% and 50% portion. This suggests that the muscle volume of the GL is smaller than that of the other muscles and less susceptible to caudal movement due to gravity. These results, taken together, suggest that it is necessary to consider the effect of posture on the measurement of the MCSA and MT, because the 50% portion of the SOL and the 25% and 50% portions of the GM are especially susceptible to postural change.

From the aforementioned results, the relationship between the MCSA and the MT is important; however, only the 50% portion of the GM was correlated, and the other sites and muscles were not. Previous studies have reported that MCSA is associated with MT [19,20]. However, these studies were conducted in the lying position, and not in the antigravity position. It is considered that the MCSA and MT were not related in the sitting position, because the muscle moved caudally and changed its morphology in three dimensions compared with the lying position. The results of this study indicate that there was a difference in the MCSA between the lying and sitting positions in the 50% portion of the GM, and that there was a correlation. Therefore, MT is considered a useful indicator of the MCSA in the sitting position. In addition, the conventional MT measured by US in the lying position may be used as an indicator of the MCSA in the sitting and lying positions because there was no difference in the MCSA between the two positions at the 25% portion of the GL and SOL. The MCSA of the 50% portion of the SOL and the 25% portion of the GM differed between the lying and sitting positions, and there was no correlation with the MT. Therefore, it would be difficult to use the MT of these sites as an indicator of MCSA in the sitting position.

One of the limitations of this study is that it only measured the MT and MCSA, which are indicators of structural factors of muscle strength, and did not directly measure muscle strength itself, or the neural factors. Some reports suggest that the MT and MCSA are correlated with muscle strength, but not strongly, and are not sufficient as direct indicators of muscle strength [22,23,24]. Therefore, the results of this study should be used only as a structural indicator. However, it is important to note that structural muscle assessment may be a more useful assessment when combined with the assessment of neurological factors such as muscle activity and fatigue by electromyography. The MT in the 50% portion of the SOL and 25% portion of the GM, which could not be used as an indicator of the MCSA in the sitting position, may be useful as an indicator of muscle strength and therefore should be investigated in the future. The number of participants in this study was only 16, which is not sufficient. Further studies with more participants are needed to verify the study findings. In addition, the MCSA measured in this study was anatomical MCSA, and physiological MCSA was not measured. Fukunaga et al. [6] suggested that physiological MCSA is necessary because anatomical MCSA alone is insufficient to assess maximal muscle strength. Therefore, the relationship between physiological MCSA and maximal muscle strength should be investigated in the future.

## 5. Conclusions

The present study investigated the differences in the MCSA of the triceps surae between the lying and sitting positions and the relationship between the MCSA and MT in the sitting position. The results indicated that the MCSA of the SOL and GM changed in the lying and sitting positions, and only the 50% portion of the GM showed a correlation between MCSA and MT in the sitting position. Therefore, it is necessary to consider the muscle and the region when the MT in the sitting position is used as an indicator of the MCSA.

## Figures and Tables

**Table 1:** Muscle cross-sectional area in the sitting and lying positions (cm^2^).

Portion	25% Portion		50% Portion		75% Portion	
Position	Sitting Position	Lying Position	Sitting Position	Lying Position	Sitting Position	Lying Position
SOL	786 ± 300	650 ± 288 ^†,#^	2260 ± 345 *	1990 ± 359 ^†,‡^	1258 ± 558	1329 ± 498 ^#^
GM	1018 ± 205 *	858 ± 230 ^†^	1333 ± 327 *	1215 ± 272 ^†,#^		
GL	655 ± 186	613 ± 193	705 ± 225	656 ± 208		

Data are presented as mean ± standard deviation. SOL, soleus; GM, gastrocnemius medial head; GL, gastrocnemius lateral head * Significant difference in the muscle cross-sectional area between the sitting and lying positions (*p* < 0.05). ^†^ Significant difference compared with the gastrocnemius lateral head (*p* < 0.05). ^‡^ Significant difference compared with the gastrocnemius medial head (*p* < 0.05). ^#^ Significant difference compared with the 50% portion (*p* < 0.05). Significant difference compared with the 75% portion (*p* < 0.05).

**Table 2:** Muscle thickness by ultrasonography in the sitting position (cm).

Portion	25% Portion	50% Portion	75% Portion
SOL	3.04 ± 0.52	3.02 ± 0.66	1.67 ± 0.90
GM	1.83 ± 0.26	0.72 ± 0.59	
GL	1.49 ± 0.19	0.25 ± 0.24	

Data are presented as mean ± standard deviation. SOL, soleus; GM, gastrocnemius medial head; GL, gastrocnemius lateral head.

**Table 3:** Muscle volume of each muscle (cm^3^).

Position	Sitting Position	Lying Position
SOL	3.64 ± 0.62	3.39 ± 0.76 *^,†^
GM	2.13 ± 0.43	2.05 ± 0.49 *
GL	1.24 ± 0.17	1.15 ± 0.15

Data are presented as mean ± standard deviation. SOL, soleus; GM, gastrocnemius medial head; GL, gastrocnemius lateral head * Significant difference compared with the gastrocnemius lateral head *(p* < 0.05). ^†^ Significant difference compared with the gastrocnemius medial head (*p* < 0.05).
